# Self-projection in middle childhood: a study on the relationship between theory of mind and episodic future thinking

**DOI:** 10.1007/s10339-021-01013-w

**Published:** 2021-02-13

**Authors:** Ines Adornetti, Alessandra Chiera, Daniela Altavilla, Valentina Deriu, Andrea Marini, Giovanni Valeri, Rita Magni, Francesco Ferretti

**Affiliations:** 1 Cosmic Lab, Department of Philosophy, Communication and Performing Arts, Roma Tre University, Via Ostiense 234, 00146 Rome, Italy; 2 Department of Languages and Literatures, Communication, Education and Society, University of Udine, Via Margreth, 3, 33100 Udine, Italy; 3 Claudiana - Landesfachhochschule Für Gesundheitsberufe, Bozen, Italy; 4 Child and Adolescent Neuropsychiatry Unit, Department of Neuroscience, The Bambino Gesù Children’s Hospital, IRCCS, Piazza Di Sant’Onofrio, 4, 00165 Rome, Italy

**Keywords:** Cognitive development, Core brain network, Episodic future thinking, Self-projection, Simulation, Theory of mind

## Abstract

Growing evidence suggests that theory of mind (ToM) and episodic future thinking (EFT) are closely related at both brain and functional level. This study explored the relationship between ToM and EFT in 96 Italian-speaking children with typical development aged between 8 and 10.11 using a behavioral design. ToM was assessed through an emotional facial expression recognition task. EFT was assessed with a task where participants were required to project themselves forward in time by anticipating future states of the self; this resulted in two scores: a nonverbal measure and a verbal explanation measure. Results showed that the participants’ performance on the task assessing ToM correlated with and predicted the nonverbal measure of the EFT task. These findings are discussed in the light of theories suggesting that each of these abilities is governed by a common system devoted to self-projection.

## Introduction

Over the last years, a significant number of investigations has provided growing evidence that abilities such as understanding the perspective of another person (Theory of Mind: ToM), remembering the past (Episodic Memory: EM), imagining the future (Episodic Future Thinking: EFT), and navigating in space (Mental Space Travel: MST) share important functional similarities and rely on a common *core brain network*. This network includes frontal and medial-temporal systems, the temporo-parietal junction, precuneus, posterior cingulate, and retrosplenial cortex (Buckner and Carroll 2007; Hassabis and Maguire 2009; Spreng et al. 2009; Spreng and Grady 2010).

A number of models have been proposed to account for the nature of this core network (e.g., Buckner and Carroll 2007; Hassabis et al. 2007; Hassabis and Maguire 2009; Schacter and Addis 2007). According to one prominent hypothesis advanced by Buckner and Carroll (2007), the four cognitive abilities associated with such network are functionally similar as rely on a process of ‘self-projection’, which is defined as a shift of perception from the immediate environment to an alternative and imagined one, with the imagined event referenced to oneself. Notably, in Buckner and Carroll’s (2007) view, the mental construction of an imagined alternative event can be conceived as a form of ‘simulation’: ToM, EM, EFT, and MST are all cases of mental simulation of, respectively, another individual’s perspective, another time, and another place.

According to the simulation-based account of ToM (i.e., the ability to attribute mental states, feelings and emotions to others), self-projection is part of mentalizing because individuals take on the perspective of others, i.e., simulate their experiences, using knowledge about their own thoughts, feelings, and preferences (putting themselves in the other person’s shoes) (Shanton and Goldman 2010; Schurz et al. 2015). EM involves simulating the past: it allows people to remember events of their own past not simply by extracting from memory a specific meaning or knowledge, but rather by re-experiencing previous past personal episodes (Tulving 1985, 2005). In this respect, remembering the past is a process of projecting the self back in time: the individual projects herself/himself in the retrieved moment reliving both details and feelings associated to that event. A large body of research has demonstrated that EM is part of a more general ability of mental time travel (MTT; Suddendorf and Corballis 1997, 2007; Michaelian 2016), which also includes EFT, namely the ability to simulate experiences that might occur in one's own personal future. Specifically, it has been suggested that individuals build alternative future perspectives on retrieved details of past experiences (autobiographical memories extracted from EM) (D’Argembeau and Mathy 2011) – although the two processes of remembering the past and imagining the future do not fully overlap (Hill and Emery 2013; Okuda et al. 2003; Schacter et al. 2012). Mental simulation also underlies some forms of spatial navigation (Chersi et al. 2013) since navigating the external environment requires individuals to imagine their own current position, the destination to be reached, and the possible routes using both allocentric (world-centered) and egocentric (person-centered) perspective (Aguirre et al. 1998).

The present study aimed to explore the possible connections between two of the abilities comprising the self-projection core network, namely ToM and EFT. Although the four components associated with this network are all deeply intertwined, it has indeed been suggested that a privileged link occurs between the social and temporal projections, with very close similarities involving the ability to take on another person’s perspective and the ability to imagine hypothetical events that might occur in one's personal future (Moore and Lemmon 2001; Suddendorf and Corballis 2007; Suddendorf and Redshaw 2013; Spreng et al. 2009; Spreng and Grady 2010). On one hand, to imagine what oneself (and others) might do in future usually requires some recognition of different mental states: when one projects oneself into a future scenario, she/he typically predicts how she/he might feel and think in that context, or what other individuals might feel and think when the simulated future event involves other people than her/himself. On the other hand, understanding others’ minds is not only a process to give meaning to current/present behavior, but a critical function is to anticipate the behavior of other individuals by recognizing their plans and goals (e.g., Berthoz 2000). From this point of view, the understanding of others’ feelings, beliefs, and thoughts is strongly intertwined with the understanding of what people might do in future.

In line with these indications, interesting similarities in the developmental trajectories of the ability to simulate the perspective of others and that of imagining one’s own future self have been reported. They both begin to develop around the age of four (Atance and Meltzoff 2005; Atance and O’Neill 2005; Callaghan et al. 2005; Suddendorf and Busby 2005; Wellman et al. 2001), show improvement during middle childhood (Schwanenflugel et al. 1998; Lecce et al. 2014; Ferretti et al. 2018a), and extend into adolescence and adulthood (Apperly 2011; Gott and Lah 2014; Wang et al. 2014). Evidence for these similarities comes also from the observation of children with neurodevelopmental disorders, such as Autism Spectrum Disorders (ASD). Accumulating evidence shows that individuals with ASD may exhibit decreased ability to project themselves into the future (Lind and Bowler 2010; Terrett et al. 2013; Marini et al. 2016, 2019; Ferretti et al. 2018b), along with deficits in ToM (Baron-Cohen et al. 1985; Happé 1994; Tager-Flusberg and Sullivan 1995; Yirmiya et al. 1998).

Notwithstanding these developmental similarities, interestingly just few behavioral studies have systematically explored the potential relation between ToM and EFT. In fact, although there is evidence for a relationship between ToM and EM (e.g., Naito 2003; Perner et al. 2007; Jarvis and Miller 2017), EM and EFT (e.g., Busby and Suddendorf 2005; Addis et al. 2007; Szpunar and McDermott 2008; Szpunar 2010; Wang et al. 2014), MTT and MST (e.g., Hasselmo 2009; Gauthier and van Wassenhove 2016), to the best of our knowledge indications about the specific association between ToM and EFT are scarce (Moore et al. 1998; Ford et al. 2012; Altgassen et al. 2014). Moore et al. (1998) examined the connections between the development of theory of mind (as well as executive functions) and the development of future-oriented prosocial (sharing) behavior in children aged from 3 to 4 years. Children were administered tests of ToM aimed at assessing their understanding of other’s beliefs and desires (i.e., the belief tasks). In addition, participants received a delay of gratification task evaluating their ability to forgo a current opportunity to get a reward (stickers) in order to gratify their own future desires or the current or future desires of another person. Results showed that ToM abilities correlated with the tendency to opt for delayed rewards in order to share with the partner in 4 years old children, but not in the youngest ones.

Subsequent investigations focused on the link between ToM and prospective memory, i.e., the ability of remembering to perform a desired action in future. According to some scholars, prospective memory involves self-projection in the form of future simulation; thus, it relies on EFT (Brewer and Marsh 2010; Neroni et al. 2014; Nigro et al. 2014). Ford et al. (2012) showed that in children between 4 and 6 years of age performance on a task assessing ToM (false-belief test) predicted success on measures of prospective memory. The authors interpreted this finding as evidence that both ToM and prospective memory involve the capacity for self-projection and that “children with a better developed theory of mind were more inclined to think about themselves performing the desired response in the future” (p. 484). This interpretation has been further corroborated by Altgassen and colleagues (2014), who showed that ToM predicted prospective memory performance also in adolescents aged between 13 and 14 years.

Nonetheless, other studies failed to find any association between ToM and prospective memory (Jarvis and Miller 2017) as well as ToM and EFT (Hanson et al. 2014). Hanson et al. (2014) investigated the relation between ToM, EFT and executive functions in preschoolers (3–4-5 years old). Analyses revealed that their performance on tasks assessing EFT did not correlate to their performance on ToM and executive functions tasks. However, it should be highlighted that Hanson et al. (2014) explicitly recognized that their findings may have been due to methodological aspects related to the different nature of the tasks employed to assess EFT and ToM. Indeed, most of the ToM tasks (including false-belief task) required children to acknowledge a conflict in perspective, i.e., understanding that other people have beliefs different from their own that do not match reality, whereas the battery of tests employed to assess EFT did not require children to explicitly represent contrasting perspectives between present and future selves. The notion of *conflicting* perspectives may represent an important factor when comparing two abilities such as ToM and EFT, and results may vary depending on whether the tasks used to assess them would involve the conflictual dimension or not. For example, it is possible that the connections between these two abilities are obscured when one of the tasks employed involves conflict whereas the other does not. On the other hand, it cannot be ruled out that, even when both the tasks employed to evaluate ToM and EFT involve the presence of forms of conflict, the links between the two abilities might take second place to the representation of conflicting perspectives.

In light of these considerations, to avoid methodological bias, the current study aimed at investigating the connections between ToM and EFT using two tests that did not require participants to represent conflicting alternative perspectives. Moreover, to avoid potential linguistic effects on children’s performance, the tasks selected were characterized by minimal linguistic demands. As for the ToM task, these methodological constraints led to exclude the tests based on the false/diverse beliefs paradigm. Therefore, it has been opted for a task in which the participants had to project into the self of another person without representing conflicting situations: the *Theory of Mind-part II* subtest from the NEPSY-II (Korkman et al. 2007). This test was developed for investigating affective ToM, i.e., the ability to understand the emotions and feelings of others, as opposed to cognitive ToM, regarding understanding of beliefs, desires, and thoughts (Shamay-Tsoory et al. 2007a, b; Duval et al. 2011; Le Bouc et al. 2012; Wang and Su 2013; Fischer et al. 2017). The focus on affective ToM represents a novelty among studies exploring the connection between ToM and EFT since the few previous investigations employed tasks assessing cognitive ToM, such as the false-belief task. As regards the EFT task, it was an adaption of the *Picture Book* developed by Atance and Meltzoff (2005) and aimed at measuring children’s ability to pre-experience personal events, drawing on their sense of a future self: children are required to imagine novel hypothetical events that could happen in their own future and anticipate possible needs of the future self.

It is worth highlighting that an aspect differentiating the current investigation compared with previous analyses concerns the focus on middle childhood. Past research concentrated mainly on the relationship between ToM and EFT in preschoolers (Moore et al. 1998), adolescents (Altgassen et al. 2014), and adults (Jarvis and Miller 2017). The available evidence on the potential relationship between the two abilities in middle childhood is scanty and inconsistent, although both ToM (Devine and Hughes 2013; Lecce et al. 2014) and EFT (Ferretti et al. 2018a; Wang et al. 2014) show significant developmental improvements over this age span. For that, in the current study, the potential relationship between ToM and EFT was explored in middle childhood, namely in a group of 96 children with typical development ranging from 8 to 10,11 years old. Consistent with models suggesting functional similarities between the two abilities (Buckner and Carroll 2007; Buckner et al. 2008) and studies documenting a correlation between them in preschoolers (Moore et al. 1998) and adolescents (Altgassen et al. 2014), we hypothesized that conceiving the viewpoint of others and envisioning the future would show a similar relationship also in middle childhood.

## Materials and methods

### Participants

Ninety-six Italian-speaking children with typical development aged between 8,00 and 10,11 years (M = 9.19; SD = 0.79; 52 females; 44 males) participated in this study. All of them had nonverbal Intelligence Quotient (IQ) level in the normal range as assessed through the Raven’s Colored Progressive Matrices (Raven, 1938; Italian standardization: Belacchi et al. 2008) (M = 105.73; SD = 12.63; Range = 80–130) (Table 1). Children were recruited in local schools. In a preliminary interview, their teachers confirmed that they had normal cognitive development, as well as average school performance. According to parents’ reports, none of them had a known history of psychiatric or neurological disorders, learning disabilities, hearing or visual loss.

**Table 1:** Descriptive analyses of the group of children

	Group (*n* = 96) M (SD) [range]
Age	9.19 (.79) [8–10.11]
Education	3rd–5th grade
Gender distribution	52 females (54%); 44 males (46%)
IQ Level	105.73 (12.63) [80–130]
ToM	6.27 (1.14) [3–8]
EFT identification	5.47 (.81) [2–6]
EFT motivation	3.76 (1.35) [0–6]
WM Digit forward	7.29 (1.65) [2–12]
WM Digit backward	4.47 (1.62) [2–9]
Selective attention	44.53 (10.04) [23–74]
Sustained attention	111.93 (18.25) [57–150]

Data are expressed as means, standard deviations, and ranges

Legend: IQ, intelligence quotient; ToM, Theory of Mind; EFT, Episodic Future Thinking; WM, Working Memory

This study was approved by the ethical committees of Roma Tre University and The Bambino Gesù Children’s Hospital in Rome. Parents signed the consent form for the participation of their children to the study and for the treatment of the data.

### Methods

The participants were tested individually at school. They were administered four tasks aimed at assessing ToM, EFT, verbal working memory, and attention skills.

ToM and EFT were assessed using two behavioral tasks with minimal linguistic demands and which do not require the representation of conflicting alternative perspectives. This choice was motivated, on the one hand, by the attempt to minimize the potential interference of narrative and language skills with the assessment of mentalizing and foresight abilities and to avoid potential methodological bias related to the processing of conflicting perspectives on the other. For these reasons, in the present study, we excluded tasks involving conflict and relying heavily on language, such as false-belief tasks and autobiographical interviews. ToM was assessed by administering the *Theory of Mind-part II* subtest from the NEPSY-II (Korkman et al. 2007), a task specifically centered on the affective component of ToM. The assessment of EFT was performed through an adaptation of the *Picture Book* task by Atance and Meltzoff (2005). As previous investigations suggested that the development of both ToM (e.g., Davis and Pratt 1995; Carlson et al. 2002) and EFT (e.g., Hill and Emery 2013; Ferretti et al. 2018a) is associated with working memory, participants were also administered the digit span memory task aimed at evaluating their working memory abilities (Wechsler 1993). Finally, the *Modified Little Bells* test (Biancardi and Stoppa 1997) was included in the study to control for possible interferences of attention difficulties.

#### Theory of mind task

The participants’ ability to infer others’ emotional states and feelings, i.e., affective ToM, was assessed through the *Theory of Mind-part II* subtest from the NEPSY-II (Korkman et al. 2007), a standardized battery for neuropsychological assessment in children aged 3 to 16 years. Participants were shown nine pictures depicting a target individual, i.e., a girl named Julia, whose face was not shown and that was engaged in different social contexts (see Table 2 for the verbal descriptions of the stimuli). Then, the children were asked to select from four options the photograph that depicted the appropriate affect of the girl in the picture. Specifically, the experimenter told participants “Show me the photo that shows how Julia feels” (see Table 2 for the verbal descriptions of the items). The first item was used as a trial. One point was assigned for each correct answer for a maximum of eight points.

**Table 2:** Verbal descriptions (not included in the original task) of the items of the *Theory of Mind-part II* subtest from the NEPSY-II (Korkman et al. 2007). Each description of the social context corresponds to a picture depicting a target individual whose face is not shown. Each verbal description of the facial affect of the target individual corresponds to photographs of a girl’s face

Social context	Facial affect of the target individual	Correct answer
Falling off a bike (*trial*)	Painful / Neutral / Smiling / Pensive	Painful
Riding a roller coaster	Anger / Annoying / Scared / Skeptical	Scared
Playing with cats	Doubtful / Fantasizing / Neutral / Playful	Playful
Hugging with friends	Surprised / Hostile / Fantasizing / Happy	Happy
Bumping into a skunk	Hostile / Distrustful / Angry / Sad	Hostile
Arguing with a friend	Distrustful / Angry / Sad / Scared	Angry
Showing an empty cookie jar to a boy	Neutral / Angry / Hostile / Doubtful	Angry
Watching a broken window while wearing a baseball glove	Worried / Angry / Neutral / Distrustful	Worried
Watching a scooter with the name Julia put on it that is broken	Fantasizing / Happy / Sad / Disgusted	Sad

#### Episodic future thinking task

The *Picture Book* task adapted from Atance and Meltzoff (2005) assessed the participants’ ability to project themselves forward in time by anticipating future states of the self, such as hunger and thirst. In order to reduce the probability that children could rely on script-based knowledge, novel scenarios for which they might have limited or no experience were used. Participants were presented, one at a time, with six scenes depicting possible destinations for a trip and designed to evoke particular physiological states of the self in relatively novel situations: a sunny desert, a river with a rocky stream, a long road in a sandy desert, a snowy landscape, a waterfall, and a mountain view. After describing the photograph, each child was asked to imagine herself/himself participating in that scenario at a future time point. Then, she/he was required to select, among three images representing three common objects, which item she/he would need to take to that place (“Which of the objects portrayed in these pictures will you need to take with you in this trip?”). For each scenario (e.g., the snowy landscape), only one “correct” item could be useful to address the potential state arising there (i.e., a coat), while a second item was completely useless and not related to the scenario (i.e., a bathing suit), and a third item was semantically primed by the scenario (i.e., ice cubes) (see Table 3). The child received one point for each item that had been correctly chosen. This represented a nonverbal measure, which we termed EFT identification score (maximum six points). After the selection of the item, the child was asked to explain why she/he had given that response (“Why will you need this in your trip?”). She/he received one point if *explicitly* referred to the anticipation of a future need (see Table 3 for examples of appropriate and inappropriate motivations). This represented a verbal explanation measure, which we termed EFT motivation score (maximum six points). It should be highlighted that in the original version of the task developed by Atance and Meltzoff (2005), participants received a score of 1 for the motivation score if they used a future referent (e.g., *going to*, *will*, *when*) and words that explicitly referred to internal feelings. In the present study, the criterion of future tense use was not employed as in Italian future states can be expressed also with present tense (e.g., “Domani vado al mare” “*Tomorrow, I go to the beach”) (see also Ferretti et al. 2018a; b). Therefore, EFT motivation score was not related to the linguistic correctness of the answers provided by the children, but to their contextual appropriateness..

**Table 3:** Scenarios, item choices, and examples of appropriate and inappropriate motivations after the identification of the correct item for the *Picture Book* task adapted from Atance and Meltzoff (2005) and aimed at assessing children’s episodic future thinking

Scenario	Distracter item	Semantically associated item	Correct Item	Examples of appropriate motivation after the identification of the correct item	Examples of inappropriate motivation after the identification of the correct item
Sunny desert	Soap	Seashell	Sunglasses	*Così mi proteggo dal sole /* In this way, I can protect myself from the sun	*Perché fa molto caldo /* Because it is very warm
River with a rocky stream	Pillow	Fish	Band-Aids	*Se casco sui sassi mi sbuccio il ginocchio e mi metto il cerotto /* If I fall on the rocks, I might skin my knee and put a Band-Aid	*Perché ci sono i sassi /* Because there are rocks
Long road in a sandy desert	Present	Plant	Water	*Perché mi servirà quando avrò sete /* Because I will need it when I’ll get thirsty	*Perché nel deserto fa molto caldo /* Because in the desert it is very warm
Snowy landscape	Bathing suit	Ice cubes	Coat	*Altrimenti mi congelo /* Otherwise I’ll be freezing	*Perché c’è la neve /* Because there is snow
Waterfall	Money	Rocks	Raincoat	*Perché mi potrei bagnare /* Because I might get wet	*Perché c’è l’acqua /* Because there is water
Mountain	Bowl	Sticks	Lunch	*Perché ho fame /* Because I’m hungry	*Il cibo è sempre importante /* Food is always important

#### Assessment of verbal working memory

Participants were administered the digit span memory task aimed at evaluating their verbal working memory (WM) abilities. This test comprises two modalities: digits forward and digits backward (Wechsler 1993). In the digit span forward, the children were required to repeat in the correct order a list of digits that the examiner had pronounced. They were given two lists of three digits, two lists of four digits, and so on, up to nine digits. If a child failed to remember both lists of a particular length, the test was discontinued. One point was assigned for each list reproduced without errors. The digit span forward score was derived from the number of lists correctly repeated by the child. The digit span backward task is identical, except for the order of repetition: the child was asked to repeat the sequence of digits in the reverse order. The digit span backward score was based on the number of lists correctly repeated by the child.

#### Assessment of attention skills

The *Modified Little Bells* test (Biancardi and Stoppa 1997) provides a measure of selective and sustained attention. The child was asked to look, one at a time, at four sheets of paper, each including drawings of several little bells scattered among other small figures. The child was required to cross out all the bells on the paper within two minutes for each sheet, although she/he did not know how much time she/he had, nor how many sheets she/he would see. A differentiation between the first thirty seconds and the remaining ninety seconds of the task was determined by requiring participants to mark the bells in red before and in blue after. This allowed the acquisition of two scores: a rapidity score measuring selective attention, which was calculated by summing up the total number of bells found per sheet in the first 30 s, and an accuracy score measuring sustained attention, which was obtained by summing up the total number of bells found on all four sheets after the two minutes.

### Statistical Analysis

Pearson’s correlation coefficient between ToM, EFT (identification and motivation), age, IQ, WM (digit forward and digit backward), attention skills (selective and sustained attention) were performed. Subsequently, multiple regression analyses with EFT (identification and motivation) as dependent variables and ToM, age, IQ, WM digit forward, WM digit backward, selective attention, and sustained attention as predictors were performed. These predictors (age, IQ, WM, selective attention) were included to ensure that the association between ToM and EFT held above and beyond the contributions of these factors.

## Results

As shown in Table 4, correlation analyses showed that ToM was positively associated with EFT identification (*r* = *0.36; p* < *0.001*). Positive correlations between ToM and age (*r* = *0.22; p* = *0.031*) as well as ToM and WM digit backward (*r* = *0.25; p* = *0.012*) were also found.

**Table 4:** Correlations between Theory of Mind (ToM), Episodic Future Thinking (EFT, identification and motivation), age, Intelligence Quotient (IQ), Working Memory (WM, digit forward and digit backward), selective and sustained attention

-	ToM	EFT Identification	EFT Motivation
ToM	–	**r = .36; p = .001**	r = .10; p = .312
Age	**r = .22; p = .031**	r = .10; p = .319	r = .17; p = .092
IQ	r = .03; p = .771	r = -−17; p = .091	r = .01; p = .944
WM Digit forward	r = .15; p = .151	r = -−01; p = .932	r = -−03; p = .774
WM Digit backward	**r = .25; p = .012**	r = -−03; p = .750	r = .03; p = .788
Selective attention	r = .04; p = .720	r = .043; p = .678	r = .02; p = .850
Sustained attention	r = .10; p = .338	r = .15; p = .154	r = .05; p = .631

No significant associations between EFT (identification and motivation) and age, IQ, WM digit forward, WM digit backward, selective, and sustained attention were found. No significant associations between ToM and IQ, WM digit forward, selective, and sustained attention were found.

As shown in Table 5, significant multiple regression model with EFT identification score as dependent variable and ToM, age, IQ, WM digit forward, WM digit backward, selective attention, and sustained attention as predictors was found (*R* = *0.46; R*^*2*^ = *0.21; R*^*2*^_*adj*_ = *0.15; F (7, 88)* = *3.35; p* = *0.003; SE* = *0.75*). ToM score (*β* = *0.37; SE* = *0.10; t(88)* = *3.72; p* < *0.001*) significantly predicted the EFT identification score; age (*β* = *0.07; SE* = *0.10; t(88)* = *0.66; p* = *0.510*), IQ (*β* = *−0.20; SE* = *0.10; t(88)* = *−1.98; p* = *0.051*), WM digit forward (*β* = *0.00; SE* = *0.10; t(88)* = *0.01; p* = *0.990*), WM digit backward (*β* = *−0.13; SE* = *0.12; t(88)* = *−1.15; p* = *0.253*), selective attention (*β* = *−0.02; SE* = *0.13; t(88)* = *0.17; p* = *0.866*), and sustained attention (*β* = *0.20; SE* = *0.13; t(88)* = *1.62; p* = *0.110*) were not significant predictors.

**Table 5:** Multiple regression analyses with Episodic Future Thinking (EFT, identification and motivation) as dependent variable and Theory of Mind (ToM), age, Intelligence Quotient (IQ), Working Memory (WM, digit forward and digit backward), selective and sustained attention as predictors

	EFT - Identification	EFT - Motivation
Multiple regression model	R = .46; R^2^ = .21; R^2^_adj_ = .15 F(7, 88) = 3.35; p = .003; SE = .75	R = .20; R^2^ = .04; R^2^_adj_ = -.03 F(7, 88) = .54; p = .804; SE = 1.38
**Predictors**		
*ToM*	β = .37; SE = .10t_(88)_ = 3.72; p < .001	β = .08; SE = .11 t_(88)_ = .75; p = .457
*Age*	β = .07; SE = .10 t_(88)_ = .66; p = .510	β = .18; SE = .11 t_(88)_ = 1.55; p = .126
*IQ*	β = -.20; SE = .10 t_(88)_ = -1.98; p = .051	β = -.01; SE = .11 t_(88)_ = -.04; p = .965
*WM* *Digit forward*	β = .00; SE = .10 t_(88)_ = .01; p = .990	β = -.06; SE = .11 t_(88)_ = -.52; p = .604
*WM* *Digit backward*	β = -.13; SE = .12 t_(88)_ = -1.15; p = .253	β = -.04; SE = .13 t_(88)_ = -.32; p = .748
*Selective attention*	β = -.02; SE = .13 t_(88)_ = -.17; p = .866	β = .00; SE = .14 t_(88)_ = .03; p = .974
*Sustained attention*	β = .20; SE = .13 t_(88)_ = 1.62; p = .110	β = .03; SE = .14 t_(88)_ = .19; p = .846

Multiple regression model with EFT motivation score as dependent variable and ToM, age, IQ, WM digit forward, WM digit backward, selective attention, and sustained attention as predictors resulted not significant (*R* = *0.20; R*^*2*^ = *0.04; R*^*2*^_*adj*_ = *−0.03; F (7, 88)* = *0.54; p* = *0.804; SE* = *1.38*) (Table 5).

## Discussion

This study aimed to explore the relationship between the ability to take on the perspective of other individuals – theory of mind – and the ability of self-projecting forward in time – episodic future thinking – in a group of children with typical development. Results revealed several links between children’s performance on tasks assessing ToM and EFT. From the correlation analyses it emerged that ToM score positively correlated with one of the two measures of EFT, namely with the identification score. In addition, controlling for age, IQ, working memory, and attention abilities, the multiple regression analyses revealed that only ToM score significantly predicted the EFT identification score.

To the best of our knowledge, this was the first behavioral study that both investigated and attested a relation between the ability to take on the perspective of another person and the ability to self-project into a future scenario in middle childhood. A previous research that explored such a relation in this age span was a brain imaging work by Fair and colleagues (2008), who found that the link between ToM and EFT (as well as EM) increases with age. Specifically, the authors showed that the neural regions forming the core network are thinly functionally connected at early school ages, i.e., 7–9 years, but later develop into a cohesive interconnected network. As for the few behavioral studies that explored the connections between ToM and EFT – or other capacities related to EFT, such as prospective memory – they focused on preschoolers (Moore et al. 1998; Ford et al. 2012; Hanson et al. 2014), adolescents (Altgassen et al. 2014), and adults (Jarvis and Miller 2017). These studies obtained contrasting results. When considering research on preschoolers, Moore et al. (1998) and Ford et al. (2012) found a relation between children performance on ToM tests and measures of the tasks assessing future-oriented cognition, whereas the investigation by Hanson et al. (2014) did not. The lack of association that has emerged from Hanson et al. (2014) research might be interpreted as evidence that shifting one’s own perspective to that of another individual is a projective process different from changing one’s own perspective into a future scenario. However, as mentioned in the Introduction, it is possible to speculate that these inconsistent findings might depend on methodological aspects related to the nature of the tasks employed to assess the two abilities. Interestingly, when a link between ToM and EFT emerged (Moore et al. 1998), both categories of tasks used in the study were designed in a way that implied the representation of conflicting alternative perspectives. In contrast, when studies failed to find correlations between ToM and EFT (Hanson et al. 2014), the tests used to evaluate the two abilities were not comparable in terms of elicitation of conflicting viewpoints (one of the tasks included conflict whereas the other did not). For example, both tests used by Moore et al. (1998), i.e., the classic false-belief task and the delay of gratification paradigm, seem to involve a dimension of conflict. As for the false-belief task, it requires children to represent conflicting mental states. Indeed, to succeed on the test, children must understand that there is a conflict concerning what they know about the world and believe about another person’s belief and the actual belief of that person. Thus, children must set aside their own beliefs and attribute to the other person mental representations that *conflict* with reality (Harwood and Farrar 2006), demonstrating to comprehend that people hold different perspectives on the world. Similarly, the delay of gratification paradigm requires children to imagine and deal with noncurrent desires in *conflicting* situations, as they are asked to choose between immediate and delayed rewards. Therefore, both the false-belief task and the delay of gratification paradigm have been claimed to rely on ability of perspective shift due to the presence of conflicting alternative viewpoints (Thompson et al. 1997, p. 201).

The representation of conflicting alternative perspectives elicited by these two tasks might account for the positive correlations between ToM and future-oriented prosocial (sharing) behavior found by Moore et al. (1998). In this respect, the degree of association between ToM and EFT might be affected by the extent to which both tasks require participants to imagine a conflicting conceptual model. This methodological issue is explicitly raised by Hanson et al. (2014), who suggested that their finding of the absence of correlation between ToM and EFT in preschoolers may depend on the different involvement of “perspective shift” elicited by the tasks employed in their study. As acknowledged by the authors (Hanson et al. 2014): “We believe that our findings are best explained by the fact that the EpF [Episodic foresight] tasks in our study did not require the same kind of perspective shift that was required by … the ToM … tasks… If there is indeed a link between these two constructs, only particular tasks from each category may serve to detect it” (p. 133–134).

On the basis of these indications, in the present investigation, we opted for excluding the involvement of “conflicting” perspectives in ToM and EFT and chose two tests that did not require children to represent conflicting conceptual models. Indeed, in the *Theory of Mind-part II* subtest from the NEPSY-II and in the *Picture Book* task children need to detach from the current self and project into the self of another person or into a future self without explicitly contrasting these different selves. In line with Moore et al. (1998) findings, our results seem to prove that task characteristics are important when investigating the relation between ToM and EFT specifically: as long as tests involving representations that are structurally similar are used, i.e., involving a “conflict” or “no conflict” dimension, then a deep link between the ability of projecting into another perspective and projecting the self forward in time emerges. Future research should take this point into consideration when exploring the possible connections between ToM and EFT.

Importantly, in the present investigation, the relation between EFT and ToM has been analyzed with reference to a specific component of ToM, namely the affective one. Several studies have indeed demonstrated that ToM forms a multidimensional construct: a cognitive ToM is used to infer others’ beliefs, intentions and desires, whereas an affective ToM involves thinking about others’ emotional states and feelings (e.g., Abu-Akel and Shamay-Tsoory 2011; Shamay-Tsoory and Aharon-Peretz 2007). Research in cognitive neuroscience tends to suggest that these two aspects of mentalizing result also in different neuroanatomical bases (e.g., Kalbe et al. 2010; Shamay-Tsoory et al. 2006). In the current study, the choice of using an affective ToM task is justified on the grounds of methodological issues discussed above. That said, since the previous studies analyzing the association between ToM and EFT focused mainly on cognitive ToM, i.e., false-belief tasks, exploring whether the affective component relates differently to EFT represents an interesting novelty. From a theoretical point of view, the focus on affective ToM is consistent with the self-projection hypothesis advanced by Buckner and Carroll (2007): in their view, along with the cognitive component, also the affective one has a prominent role given that “prospection can involve conceptual content and affective states” (p. 49).

Our results seem to confirm this hypothesis, as the association between EFT and the affective ToM has been observed. Specifically, we found that scores at the affective ToM task were associated with the EFT identification score, whereas there was not a significant association with the EFT motivation score. The fact that ToM scores correlated with the EFT identification, but not with the EFT motivation, might suggest that also the ability of self-projecting forward in time forms a multiple construct characterized by both an affective and a cognitive component. Indeed, a hypothesis that sounds plausible is that the EFT identification score, requiring anticipating possible future needs and feelings, is parallel to the ability of thinking about others’ emotional states and feelings (as confirmed by the correlations analyses), thus representing the affective constituent of EFT. Conversely, the EFT motivation score, requiring thinking more deeply on the reasons behind the choice of a certain item that could be useful to address the potential internal state arising in the given future scenario, could be considered as the analogous of the ability to infer others’ beliefs, intentions and desires, thus representing the cognitive component of EFT. Clearly, as we did not investigate the two processes of mentalizing separately, the exact relation of the affective and cognitive components of ToM with EFT cannot be specified. The analysis of possible affective and cognitive components of EFT as well as their links with affective and cognitive ToM represents a potentially relevant topic that is worth addressing in future research.

It should be acknowledged a potential limitation of the present study, namely that the EFT identification score is almost at ceiling in the sample. This fact might explain why EFT measures did not correlate with age. Indeed, the original version of the *Picture Book* task developed by Atance and Meltzoff (2005) was administered to preschoolers. However, subsequent studies showed that this task can be suitably used for the assessment of EFT skills also in middle childhood with participants with typical development (Ferretti et al. 2018a) and children with ASD, who still performed significantly lower than control participants at the age of 11 years (Ferretti et al. 2018a, b; Marini et al. 2019; Adornetti et al. 2020). Our results have indeed confirmed that the second score of the task, the EFT motivation score, was not at ceiling, thus making the *Picture Book* task as a whole a reliable measure of EFT in middle childhood, also considered that such task minimizes the linguistic demands that sometimes could interfere with the performance.

Overall, in line with previous studies (Moore et al. 1998; Ford et al. 2012), the results of the present investigation confirmed that ToM and EFT are strictly associated and that this association emerges also when the tasks employed to elicit these abilities do not require representing conflicting alternative perspectives. By providing evidence of a common simulative process underlying such abilities, our findings then contribute to support from a developmental point of view the self-projection hypothesis (Buckner and Carroll 2007). Specifically, as theory of mind turned out to be a predictor of children’s ability to self-project into the future, the present results highlight that the ability to take on the perspective of another individual might represent a scaffolding for the ability to take on the perspective of the future self. Of course, compared to the general framework of the self-projection hypothesis, our investigation provides only a partial contribution since we did not test the role of  episodic memory and mental space travel. This is a limitation of our study that highlights the need of future investigations.

## References

[CR1] Abu-Akel A, Shamay-Tsoory S (2011). Neuroanatomical and neurochemical bases of theory of mind. Neuropsychologia.

[CR2] Addis DR, Wong AT, Schacter DL (2007). Remembering the past and imagining the future: common and distinct neural substrates during event construction and elaboration. Neuropsychologia.

[CR3] Adornetti I, Chiera A, Deriu V, Altavilla D, Lucentini S, Marini A, Valeri G, Magni R, Vicari S, Ferretti F (2020). An investigation of visual narrative comprehension in children with autism spectrum disorders. Cogn Process.

[CR4] Aguirre GK, Zarahn E, D’Esposito M (1998). Neural components of Topographical representation. P Natl Acad Sci USA.

[CR5] Altgassen M, Vetter NC, Phillips LH, Akgün C, Kliegel M (2014). Theory of mind and switching predict prospective memory performance in adolescents. J Exp Child Psychol.

[CR6] Apperly I (2011). Mindreaders: the cognitive basis of “theory of mind”.

[CR7] Atance CM, Meltzoff AN (2005). My future self: Young children's ability to anticipate and explain future states. Cognitive Dev.

[CR8] Atance CM, O’Neill DK (2005). Preschoolers’ talk about future situations. First Lang.

[CR9] Baron-Cohen S, Leslie AM, Frith U (1985). Does the autistic child have a “theory of mind”. Cognition.

[CR10] Belacchi C, Scalisi TG, Cannoni E, Cornoldi C (2008). Manuale CPM coloured progressive matrices: Standardizzazione italiana.

[CR11] Berthoz A (2000). The brain's sense of movement.

[CR12] Biancardi A, Stoppa E (1997). Il test delle Campanelle modificato: una proposta per lo studio dell’attenzione in età evolutiva. Psichiatria dell'Infanzia e dell’Adolescenza.

[CR13] Brewer GA, Marsh RL (2010). On the role of episodic future simulation in encoding of prospective memories. Cogn Neurosci.

[CR14] Buckner RL, Carroll DC (2007). Self-projection and the brain. Trends cogn sci.

[CR15] Buckner RL, Andrews-Hanna JR, Schacter DL (2008) The brain's default network: Anatomy, function, and relevance to disease. In: Kingstone A, Miller MB (Eds), Annals of the New York Academy of Sciences. The year in cognitive neuroscience 2008, Blackwell Publishing. 1124; 1–38. 10.1196/annals.1440.011 18400922

[CR16] Busby J, Suddendorf T (2005). Recalling yesterday and predicting tomorrow. Cognitive Dev.

[CR17] Callaghan TC, Rochat P, Lillard A, Claux ML, Odden H, Itakura S, Tapanya S, Singh S (2005). Synchrony in the onset of mental-state reasoning: Evidence from five cultures. Psychol Sci.

[CR18] Carlson SM, Moses LJ, Breton C (2002). How specific is the relation between executive function and theory of mind? Contributions of inhibitory control and working memory. Infant Child Devel.

[CR19] Chersi F, Donnarumma F, Pezzulo G (2013). Mental imagery in the navigation domain: a computational model of sensory-motor simulation mechanisms. Adapt Behav.

[CR20] D'Argembeau A, Mathy A (2011). Tracking the construction of episodic future thoughts. J Exp Psychol Gen.

[CR21] Davis HL, Pratt C (1995). The development of children's theory of mind: The working memory explanation. Aust J Psychol.

[CR22] Devine RT, Hughes C (2013). Silent films and strange stories: Theory of mind, gender, and social experiences in middle childhood. Child dev.

[CR23] Duval C, Piolino P, Bejanin A, Eustache F, Desgranges B (2011). Age effects on different components of theory of mind. Conscious cogn.

[CR24] Fair DA, Cohen AL, Dosenbach NU, Church JA, Miezin FM, Barch DM (2008). The maturing architecture of the brain's default network. P Natl Acad Sci USA.

[CR25] Ferretti F, Chiera A, Nicchiarelli S, Adornetti I, Magni R, Vicari S, Valeri G, Marini A (2018). The development of episodic future thinking in middle childhood. Cogn process.

[CR26] Ferretti F, Adornetti I, Chiera A, Nicchiarelli S, Valeri G, Magni R, Vicari S, Marini A (2018). Time and narrative: an investigation of storytelling abilities in children with autism spectrum disorder. Front psychol.

[CR27] Fischer AL, O’Rourke N, Loken Thornton W (2017). Age differences in cognitive and affective theory of mind: Concurrent contributions of neurocognitive performance, sex, and pulse pressure. J Gerontol B Psychol Sci Soc Sci.

[CR28] Ford RM, Driscoll T, Shum D, Macaulay CE (2012). Executive and theory-of-mind contributions to event-based prospective memory in children: Exploring the self-projection hypothesis. J exp child psychol.

[CR29] Gauthier B, van Wassenhove V (2016). Cognitive mapping in mental time travel and mental space navigation. Cognition.

[CR30] Gott C, Lah S (2014). Episodic future thinking in children compared to adolescents. Child Neuropsychol.

[CR31] Gray HM, Tickle-Degnen L (2010). A meta-analysis of performance on emotion recognition tasks in Parkinson’s disease. Neuropsychology.

[CR32] Hanson LK, Atance CM, Paluck SW (2014). Is thinking about the future related to theory of mind and executive function? Not in preschoolers. J Exp Child Psychol.

[CR33] Happé FG (1994). An advanced test of theory of mind: Understanding of story characters' thoughts and feelings by able autistic, mentally handicapped, and normal children and adults. J Autism Dev Disord.

[CR34] Harwood MD, Farrar MJ (2006). Conflicting emotions: The connection between affective perspective taking and theory of mind. British J Dev Psychol.

[CR35] Hassabis D, Kumaran D, Maguire EA (2007). Using imagination to understand the neural basis of episodic memory. J Neurosci.

[CR36] Hassabis D, Maguire EA (2009). The construction system of the brain. Philos Trans R Soc Lond B Biol Sci.

[CR37] Hasselmo ME (2009). A model of episodic memory: mental time travel along encoded trajectories using grid cells. Neurobiol Learn Mem.

[CR38] Hill PF, Emery LJ (2013). Episodic future thought: Contributions from working memory. Conscious Cogn.

[CR39] Jarvis SN, Miller JK (2017). Self-projection in younger and older adults: a study of episodic memory, prospection, and theory of mind. Neuropsychol Dev Cogn B Aging Neuropsychol Cogn.

[CR40] Kalbe E, Schlegel M, Sack AT, Nowak DA, Dafotakis M, Bangard C, Brand M, Shamay-Tsoory S, Onur O, Kessler J (2010). Dissociating cognitive from affective theory of mind: a TMS study. Cortex.

[CR41] Korkman M, Kirk U, Kemp S (2007). Nepsy-II.

[CR42] Le Bouc R, Lenfant P, Delbeuck X, Ravasi L, Lebert F, Semah F, Pasquier F (2012). My belief or yours? Differential theory of mind deficits in frontotemporal dementia and Alzheimer’s disease. Brain.

[CR43] Lecce S, Bianco F, Devine RT, Hughes C, Banerjee R (2014). Promoting theory of mind during middle childhood: A training program. J Exp Child Psychol.

[CR44] Lind SE, Bowler DM (2010). Episodic memory and episodic future thinking in adults with autism. J Abnorm Psychol.

[CR45] Marini A, Ferretti F, Chiera A, Magni R, Adornetti I, Nicchiarelli S, Vicari S, Valeri G (2016). Brief report: self-based and mechanical-based future thinking in children with autism spectrum disorder. J Autism Dev Disord.

[CR46] Marini A, Ferretti F, Chiera A, Magni R, Adornetti I, Nicchiarelli S, Vicari S, Valeri G (2019). Episodic future thinking and narrative discourse generation in children with Autism Spectrum Disorders. J Neuroling.

[CR47] Michaelian K (2016). Mental Time Travel: Episodic Memory and Our Knowledge of the Personal Past.

[CR48] Miyake A, Friedman NP, Emerson MJ, Witzki AH, Howerter A, Wager TD (2000). The unity and diversity of executive functions and their contributions to complex “frontal lobe” tasks: A latent variable analysis. Cogn psychol.

[CR49] Moore C, Barresi J, Thompson C (1998). The cognitive basis of future-oriented prosocial behavior. Soc Dev.

[CR50] Moore C, Lemmon K (2001). The self in time: Developmental perspectives.

[CR51] Naito M (2003). The relationship between theory of mind and episodic memory: Evidence for the development of autonoetic consciousness. J Exp Child Psychol.

[CR52] Neroni MA, Gamboz N, Brandimonte MA (2014). Does episodic future thinking improve prospective remembering?. Conscious Cogn.

[CR53] Nigro G, Brandimonte MA, Cicogna P, Cosenza M (2014). Episodic future thinking as a predictor of children’s prospective memory. J Exp Child Psychol.

[CR54] Okuda J, Fujii T, Ohtake H, Tsukiura T, Tanji K, Suzuki K (2003). Thinking of the future and past: The roles of the frontal pole and the medial temporal lobes. Neuroimage.

[CR55] Perner J, Kloo D, Gornik E (2007). Episodic memory development: Theory of mind is part of re-experiencing experienced events. Infant Child Dev.

[CR56] Phillips LH, Channon S, Tunstall M, Hedenstrom A, Lyons K (2008). The role of working memory in decoding emotions. Emotion.

[CR57] Raven JC (1938). Progressive matrices: A perceptual test of intelligence.

[CR58] Schacter DL, Addis DR (2007). The cognitive neuroscience of constructive memory: remembering the past and imagining the future. Philos Trans R Soc Lond B Biol Sci.

[CR59] Schacter DL, Addis DR, Hassabis D, Martin VC, Spreng RN, Szpunar KK (2012). The future of memory: remembering, imagining, and the brain. Neuron.

[CR60] Schurz M, Kogler C, Scherndl T, Kronbichler M, Kühberger A (2015). Differentiating Self-projection from simulation during mentalizing: Evidence from fMRI. PLoS ONE.

[CR61] Schwanenflugel PJ, Henderson RL, Fabricius WV (1998). Developing organization of mental verbs and theory of mind in middle childhood: Evidence from extensions. Dev Psychol.

[CR62] Shamay-Tsoory SG, Aharon-Peretz J (2007). Dissociable prefrontal networks for cognitive and affective theory of mind: a lesion study. Neuropsychologia.

[CR63] Shamay-Tsoory SG, Shur S, Barcai-Goodman L, Medlovich S, Harari H, Levkovitz Y (2007). Dissociation of cognitive from affective components of theory of mind in schizophrenia. Psychiat res.

[CR64] Shamay-Tsoory SG, Shur S, Harari H, Levkovitz Y (2007). Neurocognitive basis of impaired empathy in schizophrenia. Neuropsychology.

[CR65] Shanton K, Goldman A (2010). Simulation theory. Wiley Interdiscip Rev Cogn Sci.

[CR66] Spreng RN, Mar RA, Kim AS (2009). The common neural basis of autobiographical memory, prospection, navigation, theory of mind, and the default mode: a quantitative meta-analysis. J Cogn Neurosci.

[CR67] Spreng RN, Grady CL (2010). Patterns of brain activity supporting autobiographical memory, prospection, and theory of mind, and their relationship to the default mode network. J Cogn Neurosci.

[CR68] Suddendorf T, Busby J (2005). Making decisions with the future in mind: Developmental and comparative identification of mental time travel. Learn Motiv.

[CR69] Suddendorf T, Corballis MC (1997). Mental time travel and the evolution of human mind. Genet Soc Gen Psychol Monogr.

[CR70] Suddendorf T, Corballis MC (2007). The evolution of foresight: What is mental time travel, and is it unique to humans?. Behav brain sci.

[CR71] Suddendorf T, Redshaw J (2013). The development of mental scenario building and episodic foresight. Ann N Y Acad Sci.

[CR72] Szpunar KK, McDermott KB (2008). Episodic future thought and its relation to remembering: Evidence from ratings of subjective experience. Conscious Cogn.

[CR73] Szpunar KK (2010). Episodic future thought: An emerging concept. Perspect Psychol Sci.

[CR74] Tager-Flusberg H, Sullivan K (1995). Attributing mental states to story characters: A comparison of narratives produced by autistic and mentally retarded individuals. Appl Psycholinguist.

[CR75] Terrett G, Rendell PG, Raponi-Saunders S, Henry JD, Bailey PE, Altgassen M (2013). Episodic future thinking in children with autism spectrum disorder. J Autism Dev Disord.

[CR76] Thompson C, Barresi J, Moore C (1997). The development of future-oriented prudence and altruism in preschoolers. Cogn Dev.

[CR77] Tulving E (1985). Memory and consciousness Canadian Psychol.

[CR78] Tulving E, Terrace HS, Metcalfe J (2005). Episodic memory and autonoesis: uniquely human?. The missing link in cognition: origins of self-reflective consciousness.

[CR79] Wang Z, Su Y (2013). Age-related differences in the performance of theory of mind in older adults: A dissociation of cognitive and affective components. Psychol Aging.

[CR80] Wang Q, Capous D, Koh JBK, Hou Y (2014). Past and future episodic thinking in middle childhood. J Cogn Dev.

[CR81] Wechsler D (1993). Manual for the wechsler intelligence scale for children-III.

[CR82] Wellman HM, Cross D, Watson J (2001). Meta-analysis of theory of mind development: The truth about false-belief. Child Dev.

[CR83] Yirmiya N, Erel O, Shaked M, Solomonica-Levi D (1998). Meta-analyses comparing theory of mind abilities of individuals with autism, individuals with mental retardation, and normally developing individuals. Psychol Bull.

